# Safety and Efficacy of Endoscopic Retrograde Cholangio-Pancreatography in Patients of Liver Cirrhosis: A Case-Control Study

**DOI:** 10.7759/cureus.34248

**Published:** 2023-01-26

**Authors:** Chitranshu Vashishtha, Toufik Bouchelghoum, Amita Diwaker, Ankit Bhardwaj, Manoj K Sharma, Shiv Sarin

**Affiliations:** 1 Hepatology, Institute of Liver & Biliary Sciences, New Delhi, IND; 2 Gastroenterology and Hepatology, Clinic of Gastroenterology El Eulma, Province of Setif, DZA; 3 Obstetrics and Gynaecology, Institute of Medical Sciences, Banaras Hindu University, Varanasi, IND; 4 Epidemiology and Public Health, Institute of Liver & Biliary Sciences, New Delhi, IND

**Keywords:** clinical success, bleeding, complications, cirrhosis, endoscopic retrograde cholangio-pancreatography

## Abstract

Background

With the improvement in noninvasive diagnostic imaging modalities, Endoscopic Retrograde Cholangio-Pancreatography (ERCP) has evolved into a primarily therapeutic procedure. Besides being efficacious and one of the most commonly done procedures, ERCP is also associated with a high risk of complications. However, there is a lack of studies analyzing the safety and success of ERCP in patients with liver cirrhosis. We retrospectively evaluated the outcome of ERCP in patients with cirrhosis of the liver compared to non-cirrhotic patients using the database from our institute.

Methods

Patients with liver cirrhosis who underwent ERCP from January 2010 to March 2020 were analyzed. This was a matched case-control study in which one cirrhotic patient undergoing ERCP was age and gender-matched randomly to one non-cirrhotic patient. We compared adverse events and the success rate of ERCP between cirrhotic patients and non-cirrhotic patients. The primary outcome of the study was analyzing the prevalence of procedure-related adverse events and their independent risk factors in patients of cirrhosis compared to the non-cirrhotic population.

Results

Two hundred patients were analyzed in both groups. Choledocholithiasis was the most common reason for ERCP in both groups. Mean Child-Turcotte-Pugh (CTP) score and Model for End-stage Liver Disease (MELD) score in the cirrhosis group were 9.16 ±1.78 and 19.09 ±7.06 respectively. Patients in the cirrhosis group had a significantly higher frequency of complications compared to the controls: 41 (20.5 %) versus 15 (7.5%), p < 0.01. Bleeding was the most common adverse event in both groups: 19 (9.5%) vs 6(3%). High International Normalised Ratio (INR), low platelets, and cholangitis at presentation were independently predictive of post-ERCP complications. Despite a similar technical success rate, the clinical success rate was lower in the cirrhotic than in the noncirrhotic group (83.9% versus 97.9%, p=0.006).

Conclusion

The prevalence of complications following ERCP was nearly three-fold higher in patients with cirrhosis than in non-cirrhotic patients. These events were related primarily to cholangitis, coagulopathy, and the advanced status of chronic liver disease.

## Introduction

Endoscopic Retrograde Cholangio-Pancreatography (ERCP) has been a significant technological advancement in the field of endoscopy since its inception in 1968. It has an enormous role in the management of biliary and pancreatic diseases. Hence, ERCP is one of the most commonly performed procedures for pancreatobiliary disorders [1]. Compared to routine endoscopic procedures such as esophagogastroduodenoscopy and colonoscopy, ERCP requires greater endoscopic skill and precision and can be technically difficult to perform. ERCP can also be associated with various complications with rates as high as 12% reported in noncirrhotic patients. These complications range from minor ones requiring additional hospital admission for a couple of days to life-threatening ones. The most commonly encountered ones are pancreatitis (3.1%) and bleeding (2.4%) [2]. Apart from these, it can also cause bowel perforation, cholangitis, abdominal pain, and cardiopulmonary complications. These complications can be related to technical skill or difficult anatomy of the papilla, making cannulation of the desired difficult and causing injury to the duodenum, distal bile duct, or pancreatic duct. Other causes which have been implicated are endoscopic papillotomy or sphincterotomy (making a small cut on the papilla to facilitate drainage of secretions, removal of stones, or placement of stent), comorbidities, recent anticoagulant use, injecting contrast in undrained biliary radicles or repeated contrast injections in pancreatic duct [3].

Similarly, it is well known that advanced liver disease is a significant risk factor for perioperative complications related to surgery because of liver dysfunction, portal hypertension, coagulopathy, and a higher risk of anesthesia-related complications when compared to patients without underlying liver disease [4]. However, there are few studies analyzing the safety and efficacy of ERCP in patients with liver cirrhosis. The aim of this study was to compare the prevalence of complications and success rate of ERCP in patients with liver cirrhosis and their predictors compared with non-cirrhotic controls.

## Materials and methods

We performed a retrospective matched case-control study of all consecutive patients with cirrhosis who underwent therapeutic ERCP from January 2010 to March 2020 at the Institute of Liver & Biliary Sciences, India, which is a tertiary care hospital dedicated to patients of liver and pancreaticobiliary diseases. The study was approved by our Institutional Ethics Committee (IEC) / Institutional Review Board (IRB) (No. F.37/ (1) /9/ILBS/DOA/2020/ 20217/417; dated 10.06.2020). Records of patients were retrieved from the electronic database of the hospital. The demographic data, etiology of cirrhosis, platelet count, serum creatinine, liver function tests, prothrombin time (PT) INR, Child-Turcotte-Pugh (CTP) score, Model for End-stage Liver Disease (MELD) score, indications for ERCP, procedural details and post-ERCP complications and mortality within one month of the procedure were retrieved from the hospital database. We included adult (age 18-80 years) patients with an established diagnosis of cirrhosis based on medical records, laboratory tests, imaging studies, liver elastography studies, and liver biopsy. Patients with non-cirrhotic portal hypertension, prior liver transplantation, and prior sphincterotomy were excluded. Patients with incomplete medical records were also excluded. We classified the indications for ERCP into biliary and pancreatic reasons. All the patients having a platelet count of less than 50,000 per microliter and/or PT (INR) more than 1.5 were transfused platelets and/or fresh frozen plasma prior to the procedure as per hospital protocol. Cirrhotic patients, depending on the severity of liver disease, were further subcategorized as compensated (CTP scores < 8 and MELD score < 14) and decompensated (CTP score ≥ 8 and MELD score ≥14) [3,5,6,7].

The control population was selected among non-cirrhotic patients who underwent ERCP at our Institute with available follow-up from hospital medical records, outpatient records, and telephonic interviews. Similar to cases, controls with prior sphincterotomy were also excluded. The control group was randomly selected from the hospital database who had ERCP procedures during the same time period. We matched the controls for age and gender and analyzed the same number of controls as cases (i.e., the case-control ratio was 1:1). All patients received sedation with propofol during the procedure with or without fentanyl by anesthesiologists.

Complications noted after ERCP were defined by the cotton criteria [8,9]. Post-ERCP pancreatitis was defined by suggestive symptoms of pancreatitis and serum amylase elevation at least three times the upper limit of normal after more than 24 hours of the procedure and requiring admission or prolongation of the planned admission. Bleeding secondary to ERCP was defined as symptoms of gastrointestinal bleeding or endoscopic evidence of bleeding with hemoglobin decrease by ≥ 3 g/dl. Cholangitis as a consequence of ERCP was defined as fever (≥ 38º C) and clinical features suggestive of cholangitis within 24 hours of the procedure and no other source of infection.

The primary outcome of the study was the prevalence of procedure-related complications in patients with cirrhosis compared to the non-cirrhotic population. The secondary outcome of the study was the assessment of the success rate of ERCP in patients with cirrhosis compared to the non-cirrhotic population. The success of the endoscopic procedure was assessed by both technical successes as well as clinical success. Technical success was defined as successful deep canulation of the desired duct. Clinical success was defined as at least a 75% decrease in serum total bilirubin levels from the baseline after four weeks of the ERCP procedure or improvement in cholestatic signs and symptoms after the procedure as bilirubin levels may remain elevated in patients with cirrhosis of the liver even after successful drainage of bile [10].

Statistical analysis

All statistical analyses were performed using SPSS (IBM Corp. Released 2013. IBM SPSS Statistics for Windows, Version 22.0. Armonk, NY: IBM Corp). Continuous variables were mentioned as mean and standard deviation or as median and interquartile range depending upon the homogeneity of the data. Categorical variables were mentioned as numbers and percentages. Statistical analysis was performed using the Chi-Square Test for categorical data and the student t-Test or Mann-Whitney Test for continuous data. A ‘p-value of less than 0.05 was considered significant. Univariate analysis followed by binary logistic regression analysis was performed to know the independent risk factors associated with post-ERCP complications.

## Results

**Figure 1:**
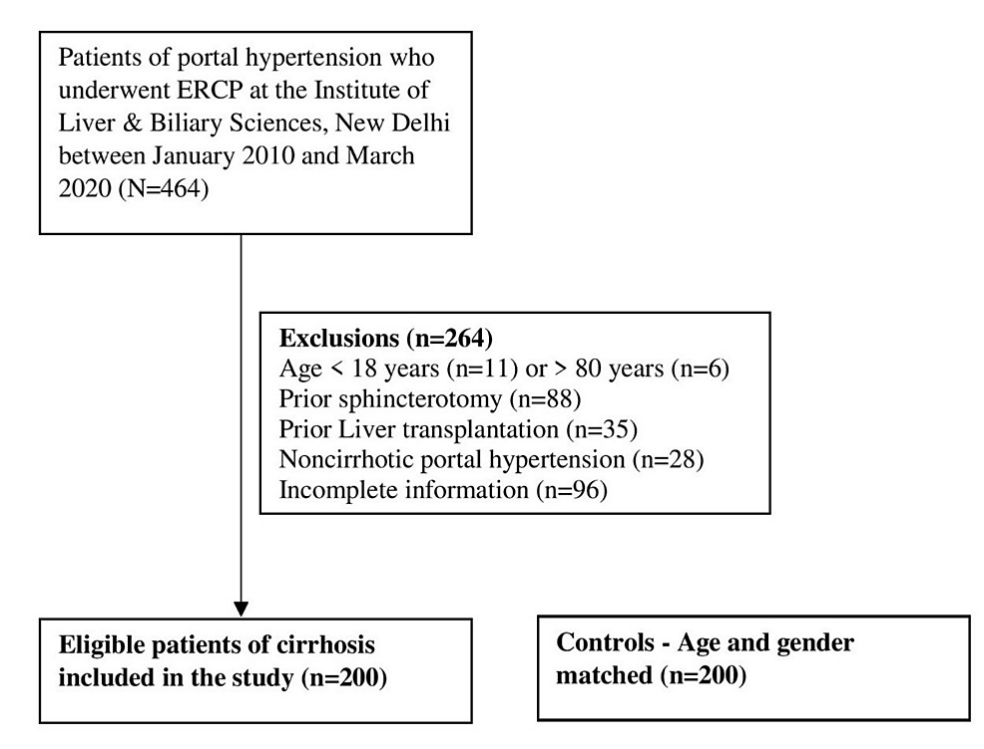
Flow chart of study participants. ERCP - Endoscopic Retrograde Cholangio-Pancreatography

During the interval from January 2010 to March 2020, a total of 464 patients with portal hypertension underwent ERCP at the Institute. Out of them, 264 patients were excluded from the analysis (age < 18 years 11, age > 80 years 6, prior sphincterotomy 88, prior liver transplantation 35, noncirrhotic portal hypertension 28, incomplete information 96). Thus, 200 patients of liver cirrhosis were analyzed in the study and they were age and gender-matched with 200 controls in 1:1 proportion (Figure 1).

The baseline demographics of the study population prior to the ERCP procedure are shown in Table 1. The mean age of the patients was 55 years and a majority of the patients were male (81.5%). Nonalcoholic steatohepatitis-related liver cirrhosis was the most common etiology of cirrhosis (24%), followed by alcohol-associated cirrhosis (23%), cryptogenic cirrhosis (14.5%) and primary sclerosing cholangitis (11.5%). Patients in the cirrhotic group had significantly lower platelet count, serum albumin levels, direct bilirubin, and higher INR compared to the controls. Cholangitis at presentation was also seen in significantly more patients in the cirrhotic group (60/200, 30%) compared to the control group (26/200, 13%), p < 0.01. Endoscopic papillotomy (EPT) was done significantly lesser in the cirrhotic group than the control group (143/200 i.e.,71.5% versus 174/200 i.e., 87%, p=0.001).

**Table 1:** Baseline characteristics of cirrhotic group and controls INR - International normalised ratio, CTP - Child-Turcotte-Pugh, MELD - Model for End-stage Liver Disease, NASH - nonalcoholic steatohepatitis, PSC - primary sclerosing cholangitis, HBV - hepatitis B virus, HCV - hepatitis V virus, HCC - hepatocellular carcinoma

Variable	Cirrhotic group (n=200)	Control group (n=200)	p value
Age (years)	55.01±13.25	54.98±13.23	0.97
Gender (male / female)	163/37	163/37	1.00
Platelet count (per µl)	140.55±91.75	276.58±106.65	0.01
INR	1.48±0.38	1.20 ±0.27	<0.01
S. creatinine (mg/dl)	0.96±0.81	0.85±0.96	0.65
S. total bilirubin (mg/dl)	8.27±8.6	8.41±9.4	0.05
S. direct bilirubin (mg/dl)	4.67±5.5	5.10±6.0	0.04
S. albumin (g/dl)	2.63±0.8	2.65±1.2	<0.01
CTP score	9.16 ±1.78	-	-
CTP class A/B/C	15/101/84 (7.5%/50.5%/42%)	-	-
MELD score	19.09 ±7.06	-	-
Etiology of cirrhosis n (%)	NASH 48 (24 %), Ethanol 46 (23%), Cryptogenic 29 (14.5%), PSC 23 (11.5%), Secondary biliary cirrhosis 20 (10%), HBV 18 (9%), HCV 13 (6.5 %), Ethanol + HBV - 3 (1.5%)		-
Indication of ERCP			
Choledocholithiasis +/-Cholelithiasis	102 (51%)	72 (36%)	
Gallbladder Carcinoma	12 (6%)	31 (15.5%)	
Carcinoma Head of Pancreas	9 (4.5%)	23 (11.5%)	
Benign Biliary Stricture	26 (13%)	4 (2%)	
Periampullary Carcinoma	10 (5%)	28 (14%)	
Acute Biliary Pancreatitis	7 (3.5%)	17 (8.5%)	
Cholangiocarcinoma	3 (1.5%)	14 (7%)	
Chronic Pancreatitis	7 (3.5%)	6 (3%)	
Obstructive HCC	12 (6%)	0 (0%)	
Portal Biliopathy	11 (5.5%)	0 (0%)	
Others (liver abscess)	1 (0.5%)	5 (2.5%)	

ERCP procedural details

Most of the procedures were related to the draining of the biliary system in both groups. ERCP procedural details were noted as biliary stenting (plastic or metallic stenting) in 179 (89.5 %) cirrhotic patient and 175 (87.5%) in non-cirrhotic patients (p=0.806). Therapeutic interventions done in the ERCP procedure in both groups are shown in Table 2.

**Table 2:** ERCP procedural details CBD - common bile duct, SEMS - self expanding metal stent

Procedural details	Cirrhotic group n (%)	Control group n (%)
Biliary plastic stent with or without CBD clearance	172 (86)	159 (79.5)
Biliary SEMS	7 (3.5)	16 (8)
No stenting	6 (3.0)	12 (6.0)
Main pancreatic duct stent	7 (3.5)	6 (3)
Technical failure	8 (4.0)	7 (3.5)
Total	200 (100)	200 (100)

Success of ERCP

There was no significant difference in the technical success rate i.e., successful cannulation of the desired duct, in both groups. The technical success rate was achieved in 192 out of 200 cirrhotics and 193 out of 200 controls, p=0.79. The clinical success rate was achieved in significantly fewer patients in the cirrhotic group versus the noncirrhotic group (83.9% in the cirrhotic group and 97.9% in the control group, p=0.006 (Table 3).

**Table 3:** Technical and clinical success

Parameter	Cirrhotic group	Control group	p value
Technical success n (%)	192/200 (96.0)	193/200 (96.5)	0.79
Clinical success n (%)	161/192 (83.9)	189/193 (97.9)	0.006

Complications related to ERCP

Patients with cirrhosis undergoing ERCP had a significantly higher frequency of complications compared to the controls: 41 (20.5 %) versus 15 (7.5%), p < 0.01 (Table 4). Bleeding was the most common adverse event seen in both groups followed by post-ERCP pancreatitis. Bleeding was mild in most cases and required admission in the high-dependency unit ward in five patients in the cirrhotic group. In the control group, out of the six patients with bleed, one was severe enough to require intensive care unit admission and interventional radiology-guided angioembolisation. All the cases of post ERCP pancreatitis were mild in severity and were successfully managed with conservative management. There was no mortality related to the ERCP procedure in both groups.

**Table 4:** Complications of ERCP in patients of cirrhosis and non-cirrhotic controls ERCP - Endoscopic Retrograde Cholangio-Pancreatography

Adverse events	Cirrhotic group, n = 200	Control group, n = 200	p value
Bleeding n (%)	19 (9.5)	6 (3.0)	0.007
Cholangitis n (%)	6 (3.0)	3 (1.5)	0.34
Post ERCP pancreatitis n (%)	8 (4.0)	4(2.0)	0.26
Duodenal perforation n (%)	1 (0.5)	1 (0.5)	1
Pneumonia n (%)	2 (1.0)	1 (0.5)	0.62
Cardiopulmonary n (%)	3 (1.5)	0	0.25
Pain n (%)	2 (1)	0	0.50
Total n (%)	41 (20.5)	15 (7.5)	<0.01

On univariate analysis, high INR, low platelet count, presence of endoscopic papillotomy (EPT), and cholangitis were predictive of complications. On binary logistic regression analysis, high INR, low platelets, and the presence of cholangitis were independently predictive of complications post-ERCP (Table 5).

**Table 5:** Univariate and Multivariate analysis for ERCP related adverse events EPT - Endoscopic papillotomy, PT (INR) - prothrombin time (international normalized ratio), OR - odds ratio

	Univariate analysis			Multivariate analysis		
Variable	OR	95% C.I.	p value	OR	95%C.I.	p value
EPT	1.16	1.007-1.390	0.05	1.04	0.4-1.7	0.61
PT (INR)	3.60	1.822-7.123	0.00	2.30	1.1-5.00	0.03
Platelet count	0.995	0.990-0.998	0.00	0.996	0.993-0.999	0.01
Cholangitis	2.34	1.273-4.302	0.00	1.96	1.02-3.72	0.04

There were significantly more cases of ERCP-related bleeding in the cirrhotic group compared to controls, 9.5% vs 3%, p=0.007. On multivariate analysis, high INR (OR 5.13,95% CI 1.86-14.12, p=0.002) and low platelet count (OR 0.99,95% CI 0.98-1.0, p=0.04) were the independent predictors of bleeding.

There was no significant difference in complications between compensated cirrhosis (defined as CPT score < 8 and MELD < 14) and the control population. There were five deaths in the cirrhosis group and two deaths in the control group, however, none of the death could be attributed to complications of ERCP.

The frequency of complications with regard to CPT score was - CPT A 0/15 (0%), CPT B 16/101 (15.8%), and CPT C 25/84 (29.8%). There was no significant difference in complications between CPT < 8 than ≥ 8, 4/34 (11.8%), and 37/166 (22.3%), p=0.17. However, there were significantly more complications in MELD ≥ 14 than < 14, 37/149 (24.8 %) and 4/51 (7.8 %), p=0.01 (Table 6).

**Table 6:** Complications in compensated and decompensated cirrhotics CTP - Child-Turcotte-Pugh, MELD - Model for End stage Liver Disease

	Proportion of patients with complications – n (%)	p value
CTP < 8 vs ≥ 8	4/34 (11.8%) vs 37/166 (22.3%)	0.17
MELD < 14 vs ≥ 14	4/51 (7.8 %) vs 37/149 (24.8 %)	0.01

**Figure 2:**
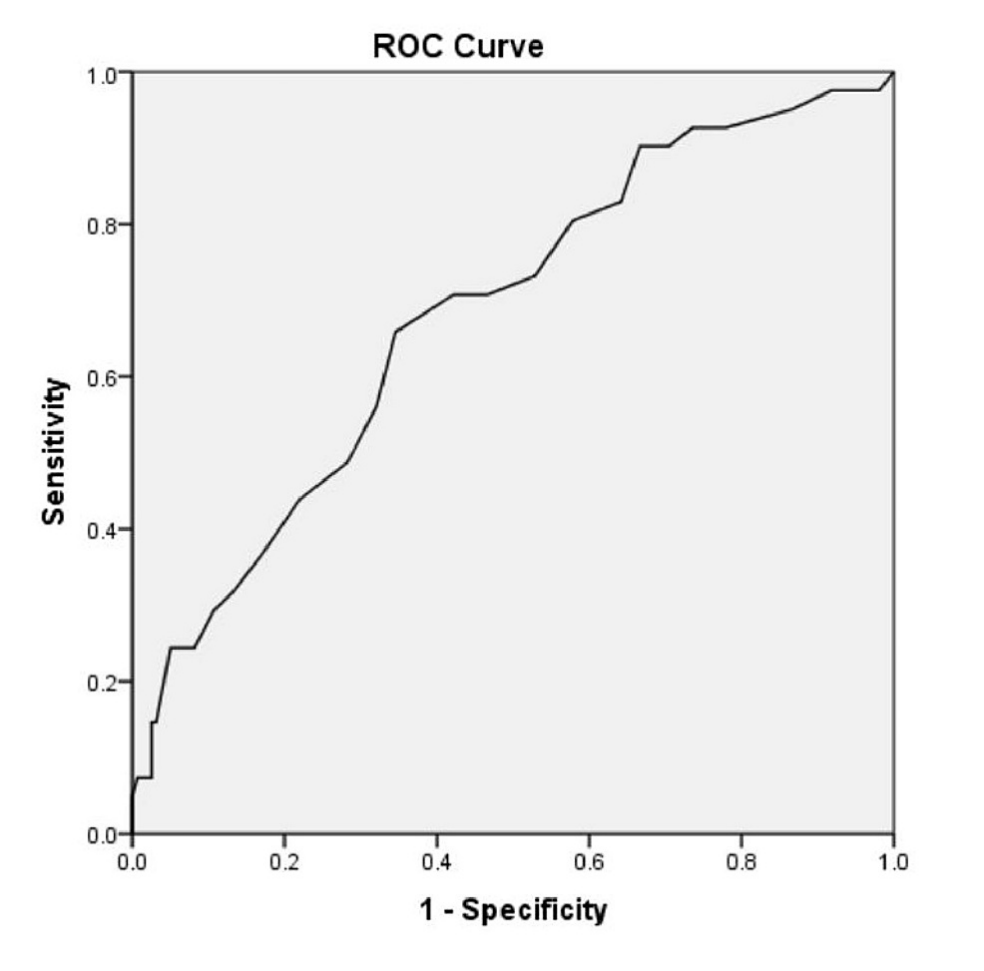
ROC showing the best cut-off to detect complications in cirrhosis according to MELD score ROC - receiver operating characteristic, MELD - Model for End stage Liver Disease

To detect the best cut-off of MELD predicting the complications related to the procedure in the cirrhotic group (n=41), receiver operating curve (ROC) analysis was done. MELD score of more than 20.5 was related to complications in this group (area under the curve 0.68, sensitivity 66 %, specificity 65 %, positive predictive value 32.9%, negative predictive value 88%, diagnostic accuracy 65.5%, positive likelihood ratio 1.9 and negative likelihood ratio 0.5, Youden Index 0.31 (Figure 2).

## Discussion

The prevalence of gallstones in patients with liver cirrhosis is high (30%, twice that in the general population) [11]. Also, cirrhotic patients can have pancreaticobiliary neoplasia similar to noncirrhotic patients, leading to obstructive jaundice. There is a potentially higher risk of ERCP-related complications in patients with liver cirrhosis. This may be due to coagulation abnormalities, higher anesthetic risk, and abnormalities in liver function which are the characteristics of patients with liver cirrhosis [10,12]. In this large, matched case-control study, we have shown that patients with advanced liver cirrhosis undergoing ERCP have a significantly higher risk of complications compared to non-cirrhotic patients. Gastrointestinal bleeding was the most common complication in these patients despite correcting with blood products prior to the procedure. This could be because routinely used tests to predict bleeding prior to an invasive procedure such as platelet count and PT (INR) correlate poorly with the bleeding risk [13]. Baseline high PT (INR) and low platelet count, suggestive of advanced liver disease, and cholangitis at the presentation predicted the adverse events in these patients. Interestingly, endoscopic papillotomy did not increase the risk of complications in patients with cirrhosis. The risk of adverse events in compensated cirrhosis was the same as in the non-cirrhotic population undergoing ERCP. Also, the MELD score of more than 20.5 was predictive of complications in the cirrhotic group with an area under the curve of 0.68, a sensitivity of 66 %, and a specificity of 65 %. The technical success rate was similar to controls in our study; however, the clinical success rate was significantly less. This could be due to the advanced nature of the liver disease in our patients as reflected by high CTP and MELD scores.

A large study from the US by Navaneethan et al., analyzed a national database that compared the adverse events and safety of inpatient ERCP between cirrhotic (n = 3228) and non-cirrhotic patients (controls, n = 12,912), showed that post-procedure bleeding was higher in patients compared to controls (2.1 % vs. 1.2 %, p < 0.01). Decompensated cirrhosis, therapeutic ERCPs, and biliary sphincterotomy were independently associated with an increased risk of post-procedure bleeding. Biliary sphincterotomy and therapeutic ERCPs increased the risk of post-ERCP pancreatitis [14]. However, patients with cirrhosis were not stratified according to the CTP score and MELD. Also, an analysis of platelet count and PT (INR) was not done in the study.

Other published studies have reported variable rates of complications after ERCP in patients with underlying cirrhosis. In a case-control study by Prat et al., analyzing 29 patients of liver cirrhosis undergoing ERCP for choledocholithiasis, endoscopic sphincterotomy was found to be safe and effective compared to controls, with no difference in morbidity and mortality [15]. However, complications in CTP class C were more serious than in classes A and B. On the other hand, a large multi-center retrospective study by Adler et al showed overall adverse events to be similar to that in a noncirrhotic population undergoing ERCP. Importantly, only 82/538 (15.2 %) patients underwent sphincterotomy during ERCP in that study. However, the prevalence of post-ERCP adverse events was higher in CTP class B and C than in CTP class A [16].

In a recently published retrospective study analyzing both the diagnostic and therapeutic ERCP in patients of liver cirrhosis from the National Inpatient Sample (NIS) Database from the United States, patients with cirrhosis had higher rates of hemorrhage (2.5% vs. 1.2%, p<0.0001), but lower bowel perforation (0.1% vs. 0.2%, p<0.0001) and pancreatitis (7% vs. 8.6%, p<0.0001) rates as compared to noncirrhotic patients undergoing ERCP. However, patients undergoing only therapeutic ERCP were not compared to the noncirrhotics, and baseline liver functions and laboratory parameters were not analyzed for predicting the complication associated with ERCP [17].

In a retrospective, multicentre matched cohort study, Leal et al. concluded that patients with cirrhosis had significantly higher adverse events compared to noncirrhotic controls, 17% vs. 9.5%, p=0.02. A novel finding of the study was that 11% of the cirrhotics developed acute on chronic liver failure after the ERCP procedure which was attributed to the ERCP procedure per se and bacterial infection. Interestingly, the complication rates were not significantly different between compensated and decompensated cirrhotics (19% vs 14.9%, p=0.42) [18]. Jagtap et al retrospectively analyzed the safety and efficacy of therapeutic ERCP in patients with cirrhosis and showed that patients with advanced liver disease as reflected by CTP class C and MELD score >18 had higher adverse events [19]. On the contrary, a study published by Ricardo Macías-Rodríguez et al concluded that the frequency of complications was not statistically different between cirrhotics and control (10% vs 8%, p=0.68). They found MELD >16 as the best cut-off for predicting post-ERCP complications [7]. 

In a recently published meta-analysis by Mashiana et al, six studies were analyzed that compared ERCP-related complications between cirrhotics and non-cirrhotic patients. Overall complication rates were higher in the cirrhotic patients with a pooled odds ratio (OR) of 1.63(95% CI: 1.27, p=0.014). Cirrhotic patients had higher rates of bleeding with OR of 2.05 (95% CI: 1.62-2.58, p<0.0001) and post-ERCP pancreatitis with OR of 1.33 (95% CI: 1.04-1.70, p=0.021), but rates of cholangitis were not significantly different with OR 1.23 (95% CI: 0.67-2.26, p=0.511). Nevertheless, the heterogeneity was high due to the marked difference in the number of cirrhotic patients analyzed in the studies [20]. Hence, the results of the studies analyzing the safety of therapeutic ERCP in patients of liver cirrhosis are heterogenous and need to be assessed prospectively.

The strength of our study is the large number of patients analyzed and compared to matched controls. The prevalence of adverse events is higher in our study compared to the studies mentioned above. This could be because our patients had a more severe liver disease as reflected by higher CTP and MELD scores in our study compared to other studies. The mean CTP score and MELD score in our study were 9 and 19.09 respectively. Compared to it, the mean CTP and MELD scores were nine and 17 in the study by Ricardo Macías-Rodríguez et al. In the study by Alder et al., only 14.8% of patients were CPT class C, whereas the mean MELD score in the study by Jagtap et al was 17.8 and in the study by Leal et al. was 10.

However, our study has several limitations. Tests utilizing global coagulation parameters in a more physiological and holistic manner such as thrombin generation test, thromboelastography (TEG), or rotational thromboelastometry (ROTEM) were not used in our patients. Also, parameters related to the risk of post-ERCP pancreatitis (number of attempts for the desired duct cannulation, contrast injection into the main pancreatic duct, use of rectal indomethacin, intravenous fluids, prophylactic main pancreatic duct stenting, and other measures to prevent post-ERCP pancreatitis) and operator specific characteristics could not be matched with controls. Our study being retrospective in nature, selection bias cannot be ruled out and hence suggests future prospective studies utilizing the tests of global hemostasis to solve the controversial issues.

## Conclusions

This study shows that ERCP has a higher risk of complications in cirrhotic patients compared to noncirrhotic patients. Low platelet count, high PT (INR), and presence of cholangitis at presentation independently predict complications related to the procedure. Biliary sphincterotomy is safe in patients with cirrhosis. However, due to the paucity of studies along with heterogeneity in the results, prospective studies evaluating the safety and clinical success of ERCP and their predictors in cirrhotic patients are needed.
